# Non-invasive imaging to monitor lupus nephritis and neuropsychiatric systemic lupus erythematosus

**DOI:** 10.12688/f1000research.6587.2

**Published:** 2015-10-27

**Authors:** Joshua M. Thurman, Natalie J. Serkova

**Affiliations:** 1 Department of Medicine, University of Colorado School of Medicine, Aurora, CO, 80045, USA; 2 Department of Anesthesiology, University of Colorado School of Medicine, Aurora, CO, 80045, USA

**Keywords:** Systemic lupus erythematosus, imaging, kidney, brain

## Abstract

Systemic lupus erythematosus (SLE) is an autoimmune disease that can affect multiple different organs, including the kidneys and central nervous system (CNS). Conventional radiological examinations in SLE patients include volumetric/ anatomical computed tomography (CT), magnetic resonance imaging (MRI) and ultrasound (US). The utility of these modalities is limited, however, due to the complexity of the disease. Furthermore, standard CT and MRI contrast agents are contraindicated in patients with renal impairment. Various radiologic methods are currently being developed to improve disease characterization in patients with SLE beyond simple anatomical endpoints. Physiological non-contrast MRI protocols have been developed to assess tissue oxygenation, glomerular filtration, renal perfusion, interstitial diffusion, and inflammation-driven fibrosis in lupus nephritis (LN) patients. For neurological symptoms, vessel size imaging (VSI, an MRI approach utilizing T2-relaxing iron oxide nanoparticles) has shown promise as a diagnostic tool. Molecular imaging probes (mostly for MRI and nuclear medicine imaging) have also been developed for diagnosing SLE with high sensitivity, and for monitoring disease activity. This paper reviews the challenges in evaluating disease activity in patients with LN and neuropsychiatric systemic lupus erythematosus (NPSLE). We describe novel MRI and positron-emission tomography (PET) molecular imaging protocols using targeted iron oxide nanoparticles and radioactive ligands, respectively, for detection of SLE-associated inflammation.

## Introduction

Systemic lupus erythematosus (SLE) is an autoimmune disease that can affect any organ throughout the body ^1^. SLE is associated with a loss of immunologic tolerance to multiple nuclear antigens and the production of autoantibodies specific for these self-antigens. The treatment of SLE almost always employs immunomodulatory therapies that suppress this autoimmune response. Immunosuppressive drugs, such as cyclophosphamide and mycophenolate mofetil (MMF), reduce tissue inflammation and injury, and the mortality for patients with SLE has improved in recent decades ^2, 3^.

SLE is a lifelong disease marked by flares and remissions. Aggressive and prolonged immunosuppression reduces - but does not eliminate - the risk of future flares. Consequently, even patients who have remained in remission for prolonged periods should continue to be monitored periodically for evidence of a disease flare. Active SLE may be clinically apparent, and several serologic tests are also helpful for monitoring disease. However, the definitive diagnosis of activity within a specific tissue requires a tissue biopsy. Unfortunately, biopsies sample only a small portion of a given tissue. In general, the implementation of repetitive biopsies in a clinical setting of immunosuppressive treatment trials remains low. Furthermore, it may not be feasible to biopsy lesions in some organs, such as the brain and spinal cord.

Radiologic assessment and qualitative imaging end-points currently have only a limited role in monitoring disease in patients with SLE. Computed tomography (CT), magnetic resonance imaging (MRI), and ultrasound (US) are frequently used to assess end organ damage in patients with specific manifestations ^4– 6^. A major limitation to the use of these studies, however, is that conventional MRI and CT contrast-agents (iodine and gadolinium based, respectively) are contraindicated in patients with renal impairment. It has become evident that for chronic inflammatory and autoimmune diseases, radiologists need tools that go beyond the standard anatomical imaging protocols. Generally, 2-deoxy-2-[ ^18^F]fluoro-d-glucose (FDG) is considered an excellent PET tracer for most inflammatory pathologies (including osteomyelitis, inflammatory bowel disease, atherosclerotic plaques) since activated granulocytes and monocytes have elevated glucose metabolism. However, antibodies can deposit in tissues prior to infiltration with granulocytes, causing inflammatory tissue injury without high ^18^FDG uptake ^7^. Hence, physiological and molecular imaging methods are being developed to detect organ dysfunctional and locate specific molecular markers in affected tissues in autoimmune diseases ^8– 11^. These methods could potentially allow clinicians to non-invasively monitor lupus disease activity.

## The unpredictable course of SLE

One of the hallmarks of lupus is that the manifestations vary between patients, and an individual patient’s disease will often vary over time. Because immunosuppressive drugs carry the risk of infection and other toxicities, the choice of treatment depends upon a patient’s specific manifestations. LN and NPSLE are two of the most severe manifestations of lupus ^3, 12– 14^. Consequently, patients who present with NPSLE or LN are frequently treated with potent immunosuppressive agents, and immunosuppression is usually continued for prolonged periods ^15, 16^.

Lupus nephritis (LN). More than 50% of patients with SLE develop renal involvement during their lives ^17^. SLE patients with LN have a higher mortality than those without renal involvement ^18, 19^, but the prognosis among patients with LN varies widely ^20^. Several histologic findings predict those patients whose disease is most likely to progress to renal failure ^17^, and this has led to the development of histologic scoring systems. The World Health Organization (WHO) classification system was published in 1982, and was revised by the International Society of Nephrology and the Renal Pathology Society (ISN/RPS) in 2004 ^21^. Class III and IV disease is characterized by inflammatory changes within the glomeruli. Glomeruli with class III and IV disease appear hypercellular and are referred to as “proliferative” LN. The prognostic value of these systems has been validated, and the ISN/RPS system is now widely employed ^21, 22^. Several clinical and laboratory findings are also of prognostic importance (such as hypertension, an elevated serum creatinine, and a low serum C3 level) ^20^, but a renal biopsy is still considered essential for deciding whether a patient requires treatment ^23^.

The standard treatment for proliferative LN involves three to six months of induction therapy with either MMF or cyclophosphamide ^24– 27^. Maintenance therapy can last for years, and the optimal duration of maintenance therapy is unknown. The response to immunosuppression is quite variable, with only ~50% of patients responding to treatment in some large trials ^24, 25, 27^. Unfortunately, most patients are treated with a “one size fits all” approach. Patients are treated according to the protocols used in the large trials, and clinicians can only determine whether a given patient will respond to treatment after months of therapy. Thus, new methods to select patients for treatment and to monitor the response to treatment are greatly needed.

Neuropsychiatric systemic lupus erythematosus (NPSLE). Up to two-thirds of patients with SLE may have some form of neurologic or psychiatric manifestations of their disease ^15, 28– 30^. Involvement of the central nervous system (CNS) can be caused by direct immune-mediated injury of tissues, systemic inflammatory mediators, vascular disease, and/or thromboembolic insults. Clinically, NPSLE has a broad range of presentations, including headaches, mood disorders, cognitive dysfunction, cerebrovascular accidents, transverse myelitis, and neuropathy ^15^. As with LN, these symptoms and findings can change over time ^31^.

Given the non-specific nature of these neurologic manifestations, it can be difficult to determine whether findings suggestive of NPSLE are caused by autoimmune mechanisms, side effects from medications, infectious complications of the disease, medications used to treat the disease, or are incidental. Obviously the CNS is less accessible for biopsy than the kidney. Consequently, the underlying pathology is less well characterized than LN. Much of the data points to vascular processes, and micro-infarcts are a common finding in autopsy series of patients with SLE ^32^. However, cerebral vascular injury may be caused by thrombotic and/or inflammatory lesions. Patients with antiphospholipid syndrome are at increased risk of stroke ^33^. Even in patients without detectable CNS lesions, antiphospholipid antibodies may be associated with cognitive abnormalities ^34^. Immunoglobulin G (IgG) and C3 deposits are detected in the brains of mice with lupus-like disease ^35^. Anti-neuronal autoantibodies have been detected in cerebrospinal fluid, and have also been detected in in brain tissue at autopsy ^36^. Overall, however, the clinical-pathologic correlation of specific CNS lesions with different clinical syndromes is unknown.

Given the wide variety of etiologies and manifestations of NPSLE, it is likely that the optimal treatment of different patients requires different approaches. The treatment of anti-phospholipid antibody syndrome, for example, primarily involves anti-coagulation. In some patients, NPSLE is likely caused by autoimmune or inflammatory factors and might effectively be treated by immunomodulatory drugs, and several case reports support this approach ^37– 40^. For patients with mild cognitive impairment, a small trial reported that prednisone may be beneficial ^41^. A randomized trial of 32 patients with severe NPSLE manifestations (e.g. optic neuritis, transverse myelitis, or coma) reported that cyclophosphamide was superior to methylprednisolone ^42^, a result similar to what is found in severe LN ^43^. Rituximab has also been effective in patients with severe NPSLE ^44, 45^. Nevertheless, little is known about which patients should be treated, what the most effective treatment is, and the optimal duration of therapy. Specific biomarkers of NPSLE would therefore make it much easier to conduct clinical trials and to identify specific patients who are likely to benefit from immunomodulatory treatment.

## The need for better biomarkers of disease activity in LN and NPSLE

Because the intensity and duration of treatment is different in patients with LN and/or NPSLE than in SLE patients who do not have renal or neurologic involvement, it is important to accurately detect involvement of these organs. The primary utility of biomarkers in this setting is to distinguish: i) patients with mild disease who do not need treatment, ii) patients with active disease who do need treatment, and iii) patients who have developed irreversible organ injury and who will not benefit from immunomodulatory treatment.

Clinically, renal involvement is detected by elevations in the urine protein excretion, erythrocytes or white blood cells in the urine, or an elevation in serum creatinine levels. Objective activity indices have also been created that incorporate these findings in order to objectively monitor patients’ renal disease and response to therapy, but these tools are more useful for clinical studies and do not replace the clinician’s assessment ^46^. As outlined above, the diagnosis of neurologic involvement is usually made on clinical grounds. The American College of Rheumatology has created classification criteria for neuropsychiatric syndromes ^47^. These definitions do not distinguish whether SLE is the underlying cause of the findings, however, and are not designed to measure disease severity.

The reactivity of particular autoantibodies has been associated with the involvement of particular organs. Unfortunately, the serologic biomarkers that are currently in use do not specifically report on lupus activity within the kidneys or CNS. Anti-double-stranded DNA antibodies ^48^, anti-ribosomal P antibodies ^49^, anti-chromatin antibodies ^50, 51^, anti-nucleosome antibodies ^52^, and decreases in C3 and C4 levels have all been associated with LN ^53^. Overall, however, the absolute levels of these markers and changes in their levels do not accurately predict a disease flare or a response to therapy. Anti-C1q antibodies also associate with LN ^54^. They have a high negative predictive value for active disease but they have a poor positive predictive value ^54^. A recent study identified alpha-enolase and annexin A1 as antigens for autoantibodies in patients with LN ^55^. Antibodies reactive against these proteins were detectable in the serum, but it is not yet known whether levels of these antibodies reflect underlying disease activity. Anti-ribosomal P antibodies ^56^, anti-glial fibrillary acidic protein antibodies ^57^, anti-N-methyl-D:-aspartate antibodies, and anti-microtubule-associated protein 2 antibodies ^58, 59^ may be associated with NPSLE. C3 and C4 levels are increased in the CSF of patients with NPSLE ^60^. Similar to the case with LN, however, the accuracy of these biomarkers for diagnosing and monitoring NPSLE has been variable in clinical studies and their role in clinical care is currently very limited ^15, 61^.

## The role of conventional radiologic imaging in LN and NPSLE

**Figure 1.**
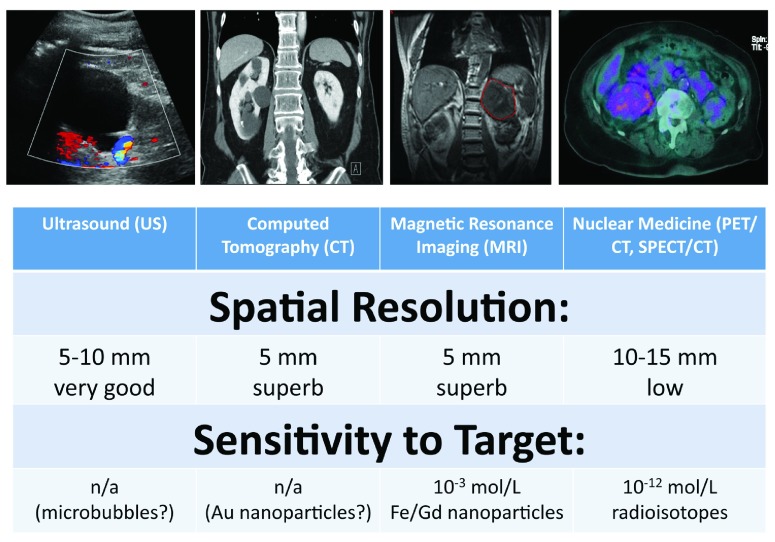
Comparison of different molecular imaging modalities. The anatomic spatial resolution and sensitivity for different molecular imaging methods are shown. CT and MRI based methods have excellent anatomic resolution, whereas PET/CT and SPECT/CT have very high sensitivity for detecting molecular targets.

Radiologic imaging (US, CT, MRI and nuclear medicine, Figure 1) has presently only a minor role in the assessment of disease activity in patients with LN or NPSLE, but possesses promising potential for future molecular assessment. CT is based on scattering and absorbing of X-ray beams while passing through the tissues; CT has a great anatomical discrimination (spatial resolution 5 mm, Figure 1) but rather a limited soft tissues contrast. It has limited application in LN patients with renal impairment since iodine-based CT contrast dyes (which are necessary due to the poor intrinsic soft tissue contrast by CT) are often contra-indicated. US sends out pulses of high-frequency sound waves and detects returning echoes scattered from the tissues. It has a very good anatomical resolution and an excellent potential for dynamic scans (Doppler). US was recently reported as a valuable platform to identify sub-clinical joint manifestations (to predict the risk of chronic deformities such as Jaccoud’s Arthropathy) in SLE patients ^6^. US is also frequently performed to examine the kidneys of patients with renal abnormalities, and it is also routinely used to guide kidney biopsies. Conventional US can detect gross changes in the kidney size and contour. Radiologically small kidneys have likely sustained chronic, irreversible changes. Decreased blood flow by Doppler might indicate irreversible disease, and it has been postulated that inflammation can initially manifest with increased blood flow. Beyond this, however, US is not useful for detecting or staging LN.

MRI is based on visualization of the physical properties of proton nuclei in tissue water in response to excitation by radio-frequency waves in a strong magnetic field. MRI has evolved as the method of choice for both LN and as well as NPSLE patient sub-populations. It has high spatial resolution (comparable to CT, Figure 1) combined with high intrinsic soft tissue contrast, allowing for non-contrast image protocols since gadolinium-based MRI contrast is also prohibited in patients with renal impairment. Even though standard MRI also has a limited role in the assessment of LN, it can detect kidney edema in patients with glomerulonephritis ^62, 63^. Most importantly, MRI allows for non-contrast physiology-based imaging which increasingly plays an important role for assessing renal function. Tissue oxygenation in the cortex and medulla has been assessed by blood-oxygenation-level-dependent (BOLD) MRI ^10^. Glomerular function can be assessed by arterial spin labeling (ASL) perfusion MRI protocols. Diffusion-weighted MRI (DWI) is based on random microscopic motion of tissue water (the Brownian motion = diffusion) which provides quantitative imaging end-points (so-called apparent diffusion coefficients, ADCs). DWI helps to characterize interstitial diffusion and to some extent renal fibrosis ^9, 10, 65^.

MRI is also the main modality used in neuroradiology. Because NPSLE is frequently caused by vascular disease (thromboembolic, hemorrhagic, or inflammatory), radiologic imaging is frequently performed in these patients. Conventional MRI and especially DWI are sensitive for the detection of strokes and transverse myelitis ^64^. A number of other CNS abnormalities detected by MRI have been reported in lupus patients, including subcortical focal lesions, cortical atrophy, ventricular dilation and cerebral edema ^65^. Some MRI findings may indicate acute, reversible processes, including diffuse, high intensity lesions, as well as hyperintensity in gray matter adjacent to the lesion and brain atrophy ^11, 15, 65^. Similar to the kidney, brain perfusion can be assessed by non-contrast ASL. More recently, a novel sophisticated MRI platform, called vessel size imaging (VSI, using commercially available T2-contrast agents, usually iron nanoparticles) has been used to precisely characterize brain vascularization, cerebral blood volume, vessel diameter, and vessel permeability ^66^. Since iron oxide contrast is not associated with toxicities in patients with renal impairment, VSI holds promise for the future. VSI has been used in pre-clinical models of stroke and oncology (particularly for gliomas) and is being recently translated into clinical oncology trials; it has not yet been applied in NPSLE or LN research.

Nuclear medicine methods include the gamma camera, positron emission tomography (PET) and single photon emission tomography (SPECT). These modalities permit *in vivo* detection of free isotopes or more complex compounds labeled with radioisotopes. Because of their low spatial resolutions (in the range of 15 mm), PET and SPECT are usually performed in combination with CT for anatomical alignment. PET is the most promising technique for molecular imaging: its sensitivity to the target lies in a picomolar range for PET-based tracers as compared to the millimolar range for MRI ( Figure 1). PET detects a decay of positron-emitting radionucleotides (such as ^18^F-, ^11^C-, ^124^I-, ^64^Cu-) by capturing a pair of gamma rays. The most commonly used PET tracer is ^18^FDG which accumulates in inflammatory cells and can be used to detect tissue inflammation. It has been used to monitor renal inflammation in a pre-clinical model ^67^, but has not been formally tested in patients with LN. ^18^FDG-PET abnormalities are very common in patients with NPSLE ^68^. These abnormalities may represent prior injury to the CNS, however, and do not distinguish active from chronic injury ^15^, thus a more specific inflammatory probe is highly desirable. The main advantages of PET is that radiolabeled proteins and peptides can be synthetized for conferring imaging visibility of targets and their activities. It can detect these markers with high sensitivity and localize the signal to specific anatomic sites, particularly when the images are co-registered with MRI or CT images.

## The promise of molecular imaging for monitoring LN and NPSLE

There is strong evidence that most of the MRI abnormalities described above are related to tissue inflammation, which is frequently present in SLE. Therefore, an idea of imaging the molecular features of SLE-driven inflammation represents an attractive and direct approach for visualizing “active” SLE. Molecular imaging is a fast developing radiological area and, without doubt, PET and SPECT are the two modalities with the highest potential in the area of molecular imaging. When using radioactive probes, both nuclear medicine techniques have higher sensitivity and specificity for targets than does MRI, and the scans are obtained relatively quickly. Importantly, routine radiolabeling protocols are available and/or can be designed rapidly. However, compared to MRI (which frequently uses targeted iron oxide nanoparticles), nuclear medicine based molecular imaging requires high dose radioactivity (especially for a slow kinetic probes such as ^124^I) and has low spatial resolution.

Several molecular imaging probes have been developed to detect markers of tissue inflammation [reviewed in 69, 70]. Pre-clinical studies have used radiolabelled antibodies against granulocytes, lymphocytes, as well as anti-TNF-alpha, anti-CD20, anti-CD2, anti-CD3, and anti-CD4 monoclonal antibodies, for both PET ( ^124^I-based) as well as SPECT imaging ( ^123^I, ^99^mTc, ^111^In) ^71, 72^. Considerable success has been reported with peptide imaging, such as radiolabelled cytokines and interleukins, as well as peptide ligands for somatostatin receptors. For the most part, these probes have not yet been tested in pre-clinical models of lupus or in lupus patients. However, many of these molecular imaging probes have the potential to detect immune proteins that deposit in affected tissues. For LN, these imaging agents and methods could enable non-invasive staging of kidney disease using these validated markers. Given the wealth of existing data regarding the deposition of immunoglobulin and complement proteins, one can infer that these molecules will likely be of diagnostic and prognostic importance. Because percutaneous renal biopsies are regularly performed, new molecular imaging probes can be compared to the biopsies in order to test the correlation of the molecular imaging method with the “gold standard” of disease staging.

Currently, the approach to patients with signs and symptoms of NPSLE is to search for underlying thromboembolic, infectious, metabolic causes, and to treat those factors ^15, 61^. Findings suggestive of antiphospholipid syndrome and/or active SLE can also inform the treatment of these patients. For NPSLE, tissue biopsies are not routine, and the decision to treat patients is based upon clinical findings. Because there is less biopsy data for comparison it is harder to foresee what molecular imaging probes that detect inflammatory markers would reveal. It is difficult to predict the extent and abundance of particular inflammatory molecules, or prognostic significance of inflammatory markers. The dearth of knowledge regarding the underlying pathology of NPSLE, however, increases the importance of developing new tools for classifying patients. It is the authors’ belief that molecular imaging methods will provide new methods for detecting and quantifying inflammation within the CNS, and could provide a means of selecting which patients to treat.

Complement C3 as an imaging target. Our own molecular imaging efforts have focused on the development of probes to detect tissue-bound C3 fragments. There are several aspects of C3 that make it particularly useful as a biomarker of SLE. First, during complement activation by immune complexes, millions of C3 molecules are cleaved and covalently fixed to nearby tissues ^73, 74^. These fragments provide a durable tissue biomarker, and biopsies from patients with SLE are usually stained for C3. Interestingly, the detection of glomerular C3 within a renal biopsy is predictive of progression of renal disease ^75^. The abundance of deposited C3 is likely to be, therefore, a sensitive and dynamic marker of inflammation. C3 is deposited in a wide range of renal diseases, however, so it is not specific for LN ^76^.

The metabolism of C3 during complement activation generates C3 fragments that remain covalently bound to tissues (C3b, iC3b, C3dg, and finally C3d). A major difficulty in developing probes to detect tissue bound C3 fragments is that the probe must distinguish the C3 fragments that are fixed to tissues from intact C3 protein in blood. During complement activation C3 undergoes conformational shifts and fragments of the protein are cleaved by proteases ^77^. Thus, there are epitopes on the cleavage fragments that are not present on intact C3. We have developed two classes of imaging probes to detect C3. We have used a recombinant form of complement receptor-2 (CR2) to bind C3 fragments. CR2 is a complement receptor expressed on B cells and follicular dendritic cells. CR2 binds the C3d cleavage fragment of C3 with a K _D_ of approximately 0.5 μM ^78^. Because CR2 does not bind intact plasma C3 it can be used to target therapeutic and diagnostic agents to sites of complement activation ^8, 79– 81^. We have also developed several monoclonal antibodies to C3d that do not bind to intact C3 or to C3b ^82^. These antibodies bind C3d with a high affinity (<1 nM) and target sites of complement activation when injected systemically ^82^.

MRI-based detection of C3 deposits. To test whether tissue C3 deposits can be detected by MRI, we conjugated recombinant CR2 to the surface of superparamagnetic iron-oxide nanoparticles (SPIONs) ^8^. Superparamagnetic iron-oxide causes rapid dephasing of nearby protons and, as a result, accelerates the spin-spin relaxation rate (R2) ^83^. Thus, T2 relaxation times decrease producing a drop in T2-weighted signal intensity (negatively enhanced T2-MRI) in areas of SPION accumulation.

We injected wild-type and lupus-prone (MRL/lpr) mice with CR2-targeted SPIONs and with untargeted SPIONs. We performed T2-weighted MRI before and after injection with the nanoparticles and analyzed the signal in the kidneys. In MRL/lpr mice, the injection of CR2-targeted SPIONs caused a significant decrease in the T2 signal within the kidneys ^8^. The T2 signal did not decrease in age-matched MRL/lpr mice injected with untargeted SPIONs, however, nor in healthy control mice injected with CR2-targeted SPIONs. These results indicate that the CR2-targeted SPIONs can be used to non-invasively detect active glomerulonephritis by T2-MRI based on tissue-bound C3-complement activation.

To determine whether this method can be used to assess disease severity, we repeated the protocol at four week intervals in MRL/lpr mice ^81^. Kidney disease worsens as MRL/lpr mice age, and the abundance of C3 fragments in the glomeruli increases until the terminal stages of the disease ^81^. The degree of negative enhancement in the kidneys of the mice increased between 12 and 20 weeks of age. These results suggest that MRI-based detection of glomerular C3 can be used to monitor the severity of the underlying disease, although this method still has limited sensitivity for detecting small differences in glomerular C3 ^81^. A study to determine whether this method can detect the response of MRL/lpr mice to immunosuppressive treatment is currently underway.

PET-based detection of C3 deposits. As outlined above, PET probes can be detected with higher sensitivity than SPIONs ( Figure 1), and we have developed high-affinity anti-C3d monoclonal antibodies that accumulate at sites of complement activation after systemic injection ^82^. Factor H deficient ( *fH ^-/-^*) mice develop spontaneous glomerulonephritis characterized by abundant glomerular C3 fragments ^84^. When injected systemically into *fH ^-/-^* mice, the anti-C3d antibodies bound to C3 fragments located within the glomeruli. We have also performed pilot PET experiments in which one of the anti-C3d mAbs was radiolabeled with ^124^I and injected into *fH ^-/-^* mice, MRL/lpr mice, and control mice, and PET/CT scans were performed 4 to 144 hrs after injection with the antibody. A high degree of signal was seen in the kidneys of *fH ^-/-^* mice and MRL/lpr mice after injection with the antibody (unpublished data).

These pilot experiments confirm that radiolabeled C3 probes can detect glomerular C3 fragments in mice with lupus-like glomerulonephritis. Future experiments will test the sensitivity of the method to distinguish mice with disease of varying severity.

## Future directions

**Figure 2.**
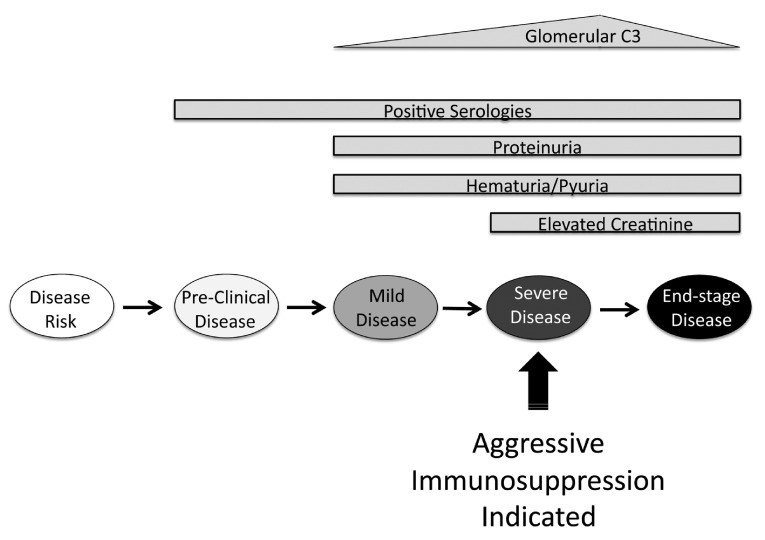
Treatment paradigm for lupus nephritis. Ideally, aggressive immunosuppression is reserved for those with severe renal disease. Lupus nephritis is associated with the presence of serologic changes, proteinuria, hematuria, and elevated serum creatinine levels. Unfortunately, these changes are not accurate for identifying patients with severe disease that is amenable to treatment. The abundance of glomerular C3 deposits increases with disease severity but falls off in end stage disease ^81^, raising the possibility that non-invasive detection of glomerular C3 will be useful for guiding treatment of patients with lupus nephritis.

The treatment of patients with SLE requires a continual reevaluation of the risks of the disease versus the risks of immunomodulatory treatment. Ideally, clinicians employ aggressive immunosuppression (e.g. cytotoxic drugs) for treating patients with severe disease, but do not use these agents in patients with mild disease or with renal damage that cannot be salvaged ( Figure 2). Currently, the assessment of lupus disease activity and prognosis is based upon a number of clinical, serologic, radiographic, and histologic findings.

LN and NPSLE are two of the most serious manifestations of SLE, and accurate assessment is critical in patients with renal or neurologic involvement. In the case of LN, tissue biopsies provide crucial information for treatment decisions, and the patterns of disease are well characterized. Unfortunately, biopsies can be subject to sampling error, and their invasive nature limits their repeated use. Molecular imaging methods may, therefore, provide a more comprehensive picture of inflammation within the kidney and will enable serial assessments as patients are treated. In the case of NPSLE, the difficulty of obtaining tissue biopsies (let alone serial tissue biopsies) is a major barrier to the full characterization of the disease process and segmentation of patients. Molecular imaging methods will enable a clearer sense of the role of inflammation in this disease, and the establishment of clinical-pathologic correlations of CNS inflammation with the broad range of neurologic symptoms that patients develop. The first clinical applications and the FDA-approval of radiotracers for detecting neurodegeneration clearly show that PET molecular imaging is feasible. The recent development of multimodality PET/MRI scanners provides the opportunity for high-resolution functional and molecular brain imaging research.

SLE is a disease that is notorious for its variable presentation and its unpredictable course. Molecular imaging biomarkers will improve our ability to care for individual patients, and our ability to evaluate the efficacy of new treatments. For individual patients, better methods of monitoring the response to therapy will allow clinicians to adjust the doses of drugs and the duration of treatment. In some cases higher treatment doses may be necessary to eliminate tissue inflammation, whereas in other patients the lower doses may be required to control tissue inflammation, and medication toxicity can be avoided.

For clinical trials, the evaluation of new drugs can take several years. Furthermore, the complex nature of SLE and the need to treat high-risk patients with established drugs has made it particularly difficult to evaluate new drugs. Treatment response is usually based on urine protein and serum creatinine measurements, and the cutoffs used to define complete and partial responses differ among trials ^24, 27, 85^. New diagnostic methods – particularly companion diagnostics for biologic therapies – will facilitate the evaluation of new drugs. Thus, new methods for detecting and monitoring inflammation within the kidneys and CNS are expected to improve the care of individual patients and to facilitate the development of new therapeutic agents. The complement system is also activated during tissue inflammation in a wide range of other diseases. Thus, the molecular imaging methods described above may also be useful for monitoring disease activity in patients with other infectious and inflammatory diseases.
