# Supplementary material for: Non-invasive imaging to monitor lupus nephritis and neuropsychiatric systemic lupus erythematosus
Source: F1000Res. 2015 Oct 27;4:153. Originally published 2015 Jun 16. [Version 2] doi: 10.12688/f1000research.6587.2 (PMC4536614; doi:10.12688/f1000research.6587.2)
Supplement: Peer review history

# Peer review: Referee response for version 1

**DOI:** 10.5256/f1000research.7073.r9535

**Published:** 2015-08-11

**Authors:** Patrick Cunningham

**Affiliations:** 1 Department of Medicine, University of Chicago, Chicago, IL, USA

This review article by Thurman and Serkova is a well-written, thorough, interesting review of the potential of newer, noninvasive imaging techniques to follow disease activity in lupus.

1. The preliminary experiments of the authors (references 8 and 80) showing the utility of this technology in mouse models is the most interesting part.  The article would benefit from much more detail of these experiments, perhaps with images if that would be illustrative.
2. However, is there any preliminary data to suggest this approach would work in the brain, as well as kidneys?
3. Similarly, more detail of the predictive power of blood tests in patients with SLE to predict outcomes would be illustrative – I suspect these biomarkers do relatively poorly.
4. Would the techniques of labeling complement, Abs, cells, etc. have applicability to other autoimmune or inflammatory diseases?
5. It is not entirely clear at first that the paramagnetic nanoparticles described on page 6 are the same as listed under MRI for figure 1.  The paramagnetic nature of the technology should be described better in the section that correlates with Figure 1.

I have read this submission. I believe that I have an appropriate level of expertise to confirm that it is of an acceptable scientific standard.

# Peer review: Referee response for version 1

**DOI:** 10.5256/f1000research.7073.r9062

**Published:** 2015-06-24

**Authors:** Dorin Bogdan Borza

**Affiliations:** 1 Meharry Medical College, Nashville, TN, USA

This well-written review article by Thurman and Serkova focuses on the utility and the potential of non-invasive imaging techniques to monitor two common yet severe manifestations of systemic lupus erythematosus (SLE), lupus nephritis (LN) and neuro-psychiatric SLE (NPSLE). The authors summarize the clinical challenges posed by the unpredictable course of SLE, explain the need for better biomarkers of disease activity in LN and NPSLE, critically review both the utility and limitations of conventional radiologic imaging, and discuss the promise of molecular imaging for monitoring LN and NPSLE.

Minor points to consider:

1. When introducing lupus nephritis (page 3), the WHO classification is mentioned only in passing. More detail will help orient the reader and provide context when discussing "proliferative LN" in the next paragraph.
2. Page 4, when discussing particular autoAbs associated with LN, the authors may consider referencing very recent studies identifying IgG2 autoAbs to alpha-enolase or annexin AI are a major component of immune deposits in kidney biopsies from LN patients ( Bruschi *et al*, 2014).
3. Page 5, C3b and C3d are introduced without providing much background information how this fragments arise.
4. On page 3, paragraph 7: NPLSE should be NPSLE.

I have read this submission. I believe that I have an appropriate level of expertise to confirm that it is of an acceptable scientific standard.

# Response

**Published:** 2015-10-17

**Authors:** Joshua Thurman

**Affiliations:** University of Colorado Denver, USA

Thank you for the thoughtful comments. We will revise the manuscript to address these points.
